# Rickettsiae in Ixodid Ticks, Sicily

**DOI:** 10.3201/eid1103.040812

**Published:** 2005-03

**Authors:** Tiziana Beninati, Claudio Genchi, Alessandra Torina, Santo Caracappa, Claudio Bandi, Nathan Lo

**Affiliations:** * Universita' degli Studi di Milano, Milano, Italy;; † Istituto Zooprofilattico della Sicilia, Palermo, Italy

**Keywords:** rickettsiae, spotted fever, Italy

**To the Editor:** Members of the spotted fever group rickettsiae are intracellular bacteria usually associated with ixodid ticks, which are transferred to vertebrates by salivary secretions and within ticks transtadially and transovarially. Several tickborne rickettsiae cause human or animal diseases and, in the last 10 years, the increased use of molecular-based identification methods has resulted in new spotted fever group rickettsiae being characterized in ixodid ticks throughout Europe (1). Until recently, no rickettsiae, other than *Rickettsia conorii*, were reported in Italy. Since 2002, *R. helvetica* and Israeli spotted fever *Rickettsia* (*R. conorii* complex) have been detected in *Ixodes ricinus* and *Rhipicephalus sanguineus*, respectively (2–4). In Italy, Mediterranean spotted fever is endemic. This disease appears to occur more commonly in some central and southern regions (5); in 2002, more than half (498 of 890) of the cases of Mediterranean spotted fever identified in Italy and reported to the Ministry of Health came from Sicily.

Because of the relatively high prevalence of rickettsial diseases in southern regions, we analyzed tick samples collected during 2001 and 2002 from herbivores (bovines, ovines, donkeys) from Sicilian farms in Corleone (Palermo Province) to determine the diversity of spotted fever group rickettsiae in various ixodid tick species. DNA from 238 tick samples from various genera (*Dermacentor*, *Rhipicephalus*, *Hyalomma*, *Haemaphysalis*, *Ixodes*) was extracted; in some cases, individual ticks of the same species collected from the same animal were pooled. Polymerase chain reaction screening and sequencing with primers for the gene encoding the cell surface antigen (*sca*4) (previously known as "gene D") and the 17-kDa antigen gene were performed as previously reported (6,7). A total of 7 positives were found, and the sequences obtained were compared to other bacterial sequences present in the GenBank database (Table). A 469-bp fragment with 100% identity to the *R. slovaca sca4* sequence (AF155054) was obtained from 2 *Dermacentor marginatus* and 1 *Haemaphysalis punctata*. A 403-bp fragment with 99.75% identity (1-bp difference) to the *sca*4 sequence from *R. africae* (AF151724) was found from 1 *Hyalomma marginatum*, and a 423-bp fragment with 100% similarity to *R. conorii sca*4 sequence (AE008626) was found from *Rhipicephalus turanicus*. Finally, a 489-bp fragment with 99.79% identity (1-bp difference) to *R. aeschlimannii sca4* sequence (AF163006) was obtained from 2 *H. marginatum* samples. The levels of identity between the 17-kDa antigen sequences (ranging in length from 351 to 419 bp) obtained during this study and those in GenBank were generally lower than those for *sca*4 because the 17-kDa antigen gene has not been sequenced for most of the *Rickettsia* spp. identified here on the basis of *sca*4 sequences. One exception was the fragment obtained from the *Rh. turanicus* sample, which had 100% identity with the *R. conorii* 17-kDa antigen sequence (AE008675). All *sca*4 and 17-kDa antigen gene sequences described in this study have been deposited in the EMBL database (accession no. AJ781411–AJ781420).

**Table:** Identification of *Rickettsia* spp. in tick samples from herbivores, Corleone (Palermo Province), Italy, 2001-2002

No. of ticks infected/total no. of ticks examined (tick species)	Minimum-maximum infection rate (%)	*Rickettsia* spp. identified (% identity with *sca*4 of spotted fever group rickettsiae)
*Dermacentor marginatus* (2/7)	28.5	*R. slovaca* (100)
*Haemaphysalis punctata* (1/15)	6.6	*R. slovaca* (100)
*Hyalomma marginatum* (2*/24)	8.3–20.8	*R. aeschlimannii* (99.79)
*Hyalomma marginatum* (1/24)	4.1	*R. africae* (99.75) *Rickettsia* sp. strain S (99.25) *R. honei* (99.0)
*Rhipicephalus turanicus* (1†/52)	1.9–7.6	*R. conorii* (100)

*Two pools of 2 and 3 ticks were positive. †One pool of 4 ticks was positive.

Among the numerous *rickettsia* species recently described in Europe, *R. africae* and *R. slovaca* are known as human pathogens (8), and the first case of *R. aeschlimannii* infection in humans has recently been reported (9). African tick bite fever caused by *R. africae* is known as an imported disease in patients returning from sub-Saharan Africa or the West Indies (8), but our report raises the possibility that the rickettsial agent is actually present in European ticks from genera other than *Amblyomma*. *R. slovaca* has been shown to be responsible for a human disease known as tick-borne lymphadenopathy (10). Considering the problem of cross-reaction between different spotted fever group rickettsiae during serologic tests, our findings underscore the importance of using antigens from other spotted fever group rickettsiae, in addition to that of *R. conorii*, to obtain a more specific diagnosis of rickettsioses in Italy (10). Considering the large number of tick species present in Italy, and their infection with different spotted fever group rickettsiae, identifying the tick species responsible for a bite could be helpful for accurate diagnosis.
